# Vaccination ethics

**DOI:** 10.1093/bmb/ldaa036

**Published:** 2020-12-26

**Authors:** Alberto Giubilini

**Affiliations:** Oxford Uehiro Centre for Practical Ethics and Wellcome Centre for Ethics and Humanities, University of Oxford, 16-17 St Ebbes St, Oxford OX1 1PT, UK

**Keywords:** vaccination ethics, public health ethics

## Abstract

Vaccination decisions and policies present tensions between individual rights and the moral duty to contribute to harm prevention. This article focuses on ethical issues around vaccination behaviour and policies. It will not cover ethical issues around vaccination research.

**Sources of data:**

Literature on ethics of vaccination decisions and policies.

**Areas of agreement:**

Individuals have a moral responsibility to vaccinate, at least against certain infectious diseases in certain circumstances.

**Areas of controversy:**

Some argue that non-coercive measures are ethically preferable unless there are situations of emergency. Others hold that coercive measures are ethically justified even in absence of emergencies.

**Growing points:**

Conscientious objection to vaccination is becoming a major area of discussion.

**Areas timely for developing research:**

The relationship between individual, collective and institutional responsibilities to contribute to the public good of herd immunity will be a major point of discussion**,** particularly with regard to the COVID-19 vaccine.

## Introduction

The coronavirus disease 2019 (COVID-19) pandemic is only the latest example of how devastating infectious diseases can be when we do not have a vaccine against them. Millions of lives are at risks today and more than a million people have died worldwide because of this virus. Millions of people died during the ‘Black Death’ (the Bubonic plague epidemic) in Europe in the 17th Century, which killed one-third of the European population—about 25 million people—over a 5 year period.^1^ More recently, the Ebola epidemic caused almost 30 000 deaths in West Africa over a 2 year period between 2014 and 2016.^2^ All these cases vary with regard to historical, geographical and socio-economic implications. The one thing they all have in common is that there was no specific vaccine available when the outbreaks started.

If we had had a vaccine, one would be inclined to think, there would be no COVID-19 pandemic, no lockdown would have been necessary, far fewer people would have died, the economy would not have been devastated and the physical and mental well-being of many people would not have been compromised. Right now, a future COVID-19 vaccine is widely seen as the end of a nightmare.

This is only partially true. If we had had a vaccine, and when we do have a vaccine that is widely available, COVID-19 will stop being a threat only if enough people are willing and given the opportunity to be vaccinated. This is where the most interesting ethical issues around vaccination policies and vaccination behaviours arise. To the extent that vaccination decisions affect not only (or even not primarily) the individuals who get vaccinated, but also people around them and the community more broadly, vaccination decisions and policies are also ethical decisions. In its most basic understanding, ethics is about values and principles that ought to regulate behaviours that are not only or not exclusively in one’s own self-interest, but also and sometimes primarily in other peoples’ interest or in line with certain societal expectations or requirements.

Looking at the trends in uptake of various vaccines in recent years, there are some reasons for concern. For example, recent drops in vaccination uptake caused measles cases to triple in Europe in 2018/19.^3^ Measles is a good example of an infectious disease where the vaccine makes a huge difference, both where it is widely available and is preventing a significant number of deaths, and where it is not widely available and people, especially children, keep dying or suffering permanent damages because of measles. For instance, the WHO estimates that the measles vaccine has saved 17.1 million lives worldwide between 2000 and 2015.^4^ One might wonder how comes that vaccination is an ethical issue at all. Given how beneficial it is and how low risk it is, and given that the benefits accrue not only to the individual who is vaccinated but to third parties and the wider community as well, one might think that there really should not be any significant ethical issue raised by vaccination.

And yet, many people do not vaccinate against measles and other common infectious diseases. Low and inconsistent vaccination uptake characterizes both low- and middle-income countries (LMICs) that do not have adequate access to vaccines^5^ but also, perhaps surprisingly, high-income countries (HICs), though for different reasons. These include fears of iatrogenic diseases,^6^^,^^7^ preferences for natural lifestyles,^8^ religious opposition to vaccines^6^ or increased sense of responsibility for the small risks of vaccines, as opposed to the responsibility for the risks of exposing one’s children to infectious diseases.^9^ Some people are simply ‘vaccine hesitant’: they do not reject vaccination tout court or in principle, but their scepticism and concerns often cause them to delay vaccination for their children.^10^

Significant drops in vaccination uptake are more likely to occur in countries with no mandatory vaccination policies or where vaccination mandates have very flexible non-medical exemption clauses, such as in many US states. Even when vaccination uptake is sufficiently high, the risk of drops in uptake always looms large. For example, in the UK, where no vaccination is mandatory, the child uptake for the MMR vaccine has declined in each of the past 5 years and has consistently been below the WHO recommended threshold of 95%, which is necessary for herd immunity.^11^

Thus, even when vaccines are widely available and when many people do vaccinate, vaccination still raises a number of ethical issues both about public policies and individual behaviours. For example, is it unethical not to be vaccinated or not to vaccinate one’s children against certain infectious diseases? What policies ought a state to implement to promote vaccination uptake? What kinds of penalties, if any, should there be for non-vaccination? How do the small risks of vaccines affect the ethics of individual vaccination decisions and of vaccination policies? Are there any groups that have a special moral obligation to be vaccinated or that vaccination policies should specifically target? There is a debate as to which vaccines exactly these questions and their answers apply. However, the questions are normally taken to be more pressing with regard to those vaccines that protect against the infectious diseases that represent major threats in certain areas, either because they are more severe or because they are more infectious, or both. For example, in most HICs the questions apply to the MMR vaccine, but not to the fever vaccine, because yellow fever is not a public health threat in those countries.

Broadly speaking, there are two main ethical principles that can ground individual, collective and institutional responsibilities for vaccination: harm prevention and fairness in the contribution to public goods. There are different ethical views on the ethical weight of both and on the extent to which they ground individual responsibilities and justify different kinds of vaccination policies. Such principles need to be weighed against countervailing considerations, such as the fact that certain people have personal views against vaccination or that vaccine do present some risk of iatrogenic harm, and countervailing principles, such as freedom of conscience, a principle of least restrictive alternative in public health, and the minimization of risks on individuals in the pursuit of the collective good. Before addressing them in more detail, it useful to say something about the relevant ethical aspects of the notion of herd immunity and about the principle of least restrictive alternative in public health.

## Herd immunity as a public good

Herd immunity (which is sometimes also referred to as ‘herd protection’ or as ‘community protection’, to emphasize its being a goal of a moral community) is the situation where enough people in a community are immune from a certain infectious disease and therefore those who are not vaccinated are indirectly protected because the high immunization rate stops the virus transmission.^12^ The threshold for herd immunity for any disease is determined both by the infectiousness of the pathogen and the effectiveness of the vaccine. For measles, for example, the threshold for herd immunity is 95% immunity, but for other infectious diseases the threshold is typically lower. Achieving and maintaining herd immunity is vital for that fraction of a population that cannot be vaccinated for medical reasons (e.g. because they are immunosuppressed) or for age reasons (e.g. the first shot of the MMR vaccine is not recommended for children before the age of 6 months). When collective immunity drops, the risk of measles outbreaks becomes higher.

From an ethical point of view, the relevant aspect of herd immunity is that it is a public good.^13^ Public goods are goods that are non-excludable and non-rivalrous in consumption. In few words, this means that it is difficult to prevent people from benefitting from them, and that individuals benefit from them regardless of how many other individuals do. For example, clean air is a public good. In the case of herd immunity, the benefit can be either direct, for those who are not vaccinated or immune, or indirect, for those who benefit from living in a community with a good level of public health and low risks of outbreaks. By their own nature, public goods raise distinctive ethical issues around fairness and free-riding. Because they are non-excludable, people would benefit from them even if they do not contribute, so there is an incentive to free-ride. Free-riding is normally considered unethical and is often illegal (e.g. in the case mandatory ticket fares for public transport, or taxation policies), because it violates fairness. And because they are non-rivalrous, the fact that someone does not contribute does not deprive others of the good—for instance, in the case of vaccination, the fact that I do not contribute to herd immunity is very unlikely to compromise herd immunity and therefore deprive others of this good. Again, at least according to some accounts, failing to make one’s contribution to herd immunity just because it doesn’t make a difference violates a fairness requirement.^14^^,^^15^

Infectious disease outbreaks can be very disruptive of normal life and can have a huge economic cost. The COVID-19 outbreak is an obvious example at the moment. It is very easy to see how vaccine-induced herd immunity against COVID-19 would be one of the most important public goods we could have right now. The realistic possibility of other pandemics raises important question about whether we have a moral obligation, and indeed we should have a legal obligation, to contribute to the realization of herd immunity when we do have a vaccine that can prevent or stop them.^16^ Indeed, even seasonal flu outbreaks, which do not reach pandemic levels, often entail a large collective cost, even for those who do not get the flu. For instance, it has been estimated that the economic impact of annual influenza epidemics in the USA, taking into account loss of earning, would amount to US$87.1 billion.^17^ As we shall see, the nature of public good of herd immunity gives rise to some distinctive ethical issues both with regard to individual behaviour and vaccination policies.

## The principle of least restrictive alternative and vaccination policies

Many would agree that Governments, to the extent that they have an obligation to protect the health of the population and the most important public goods, should try to achieve herd immunity against certain infectious diseases. The real ethical question is not *if*, but *how* and within what limits they should doit.

A widely accepted principle in public health ethics^18^ is the so called ‘principle of least restrictive alternative. There are many formulations of it in the literature. For the purpose of this review, we can take it to prescribe the implementation of the policy, among those who can successfully achieve a certain public health goal, that entails ‘the least intrusion on personal rights and freedoms’.^19^ In the case of vaccination, the rights and freedoms at stake are the right to make decisions about one’s own health and the health of one’s children, and the right to bodily integrity. The focus on rights and freedoms is however just one way of interpreting restrictiveness. An alternative interpretation of what counts as more or less restrictive might place more emphasis on the risk of harm entailed by certain public policies, rather than on liberty infringement^20^: on this understanding, a policy is more restrictive than alternative ones if it poses higher risks on the population, quite independently of the fact that it restrict liberties. In the case of vaccination, the least restrictive alternative could then also be understood as the alternative that imposes the lowest risk of harm possible at the population level, compatibly with achieving herd immunity.

If we take the first understanding of restrictiveness, then the principle of least restrictive alternative would require to implement the policy characterized by the lowest degree of coerciveness possible, and preferably a non-coercive policy, if that would allow to achieve herd immunity. On the second understanding of restrictiveness, the principle of least restrictive alternative would prescribe the implementation of the policy that would allow to immunize the minimum number of people required for herd immunity. The two requirements might or might not coincide in practice, but are conceptually distinct.

There is a range of possible vaccination policies that can be ranked in terms of restrictiveness. These go from mere information campaigns to outright compulsion or even forced vaccination. Alternatives within this range include nudging policies (e.g. opt-out vaccination in schools^21^), incentives and certain penalties for non-vaccination (e.g. not allowing non-vaccinated children in school, such as in the USA, or withdrawing certain state benefits from families who do not vaccinate their children, such as in Australia.^22–24^ Compulsory vaccination can be defined as a more extreme measure where non-vaccination is made illegal, and therefore there are legal penalties (e.g. fines) for non-compliance with vaccination requirements. For instance, Italy recently introduced a 500Euros fine for parents of non-vaccinated school age children, which turned out to be quite effective at raising MMR vaccine uptake in the pediatric population.

The principle of least restrictive alternative can be seen as a utilitarian principle (utilitarianism is the ethical theory that prescribes the maximization of expected utility,), as it aims at promoting the collective good but also at preserving as much freedom as possible. To the extent that both protection from diseases and liberty contribute to well-being, the expected utility would be maximized.

Thus, for instance, the principle of least restrictive alternative might require implementing only nudging policies, e.g. in the form of opt-out vaccination systems. As per Thaler and Sunstein’s ^25^ standard characterization, a nudge ‘alters people’s behaviour in a predictable way without forbidding any option or significantly changing their economic incentives’. Nudges might be seen to undermine autonomy to a certain degree because they might circumvent people’s capacity for rational thinking by making a certain default more prominent. However, they are not coercive to the extent that individuals are free to opt out and the degree of autonomy infringement involved is lesser than in the case of, say, mandatory vaccination. Navin and Largent^26^ have suggested that an alternative option would be to make vaccine refusal difficult—e.g. to have burdensome bureaucratic procedures for opting out of vaccination mandates, or to require parents who do not want to vaccinate their children to attend meetings where they learn about the benefits of vaccine. They do not refer to this option as a form of ‘nudge’. However, we could interpret the notion of incentives and disincentives more broadly than in the case of the definition provided by Thaler and Sunstein, which only refers to economic incentives. If we consider also practical and not only economic aspects of incentives and disincentives, then Navin and Largent’s suggestion would also count as a form of nudging policy.

Incentives are another example of policy that, according to the principle of least restrictive alternative, should be prioritized over compulsion. There is conflicting evidence as to whether incentives are effective at increasing vaccination uptake, both in children^27^ and adults.^28^ From an ethical point of view, incentives for vaccination raise two kinds of issues. First, we should ask whether they can be considered coercive. According to some philosophical understandings of coercion,^29^^,^^30^ but not according to others,^31^ large incentives can be coercive if the offers are irresistible to refuse. However, even assuming they are coercive, they seem to be less coercive than large threats of penalties. Second, regardless of whether incentives are coercive, there is an ethical issue on whether it is justifiable to pay people to do something they have an independent moral obligation to do. If vaccination is an individual moral obligation, some argue there is a stronger ethical reason to create disincentives (e.g. legal penalties) for those who fail to vaccination than to incentivize people to vaccinate.^32^

Thus, one might argue, if nudging or incentives would allow to achieve herd immunity, the principle of least restrictive alternative implies that mandatory vaccination is not justified.

However, as the incentives example shows, the principle of least restrictive alternative and its application is less intuitive than it might initiallyseem.

First, it only focusses on the balance between restrictiveness and effectiveness, but it disregards other relevant considerations, such as desert (people who ought to vaccinate as a moral obligation might not ‘deserve’ to be paid to vaccinate). Second, even if a certain policy is effective at any one time at achieving herd immunity, we have no guarantee that it will be in the future, and perhaps it is better to err on the side of safety and have measures that are stricter than necessary, just to prevent future drops in vaccine uptake. Third, the application of the principle in the vaccination case can be challenged on the basis of aiming at the wrong target: some might argue that we do not only want herd immunity to be realized, but we want everyone to contribute to it for the sake of fairness. I will say something more about this in the ‘fairness’ section below. Thus, all in all, one criticism to the principle of least restrictive alternative could be posed in terms of putting too much emphasis on the value of individual liberty, to the detriment of other considerations and principles.

## Harm prevention

Arguments for compulsory vaccination on grounds of harm prevention are not uncommon, but, because they typically take the principle of least restrictive alternative very seriously, they are often limited to cases in which there is no herd immunity and therefore where any non-vaccinated individual poses a serious risk of harm to other individuals (either vaccinated or non-vaccinated ones, since no vaccine is 100% effective).^33–36^ Otherwise, some argue, imposing on individuals the however small risk entailed by vaccines for no real benefit to oneself or to others is not justified,^13^ and neither is restricting liberty for no real benefit^34–36^.

Vaccination can be mandated—or, less controversially, can be seen as a moral obligation—either to prevent harm to the vaccinated individual or to prevent harm to others, orboth.

The first line of argument is more powerful when it comes to vaccinating children who cannot legally consent to medical interventions. It is commonly accepted that in these cases children have a right to be protected from preventable harm, which implies a right to preventive medicine.^16^ Arguably, it is a parents’ and a state’s responsibility to take reasonable steps to ensure that children’s health is protected. The argument is weaker when it comes to adult vaccination, since competent adults have a prima facie right to make their own autonomous decisions about their health and the risks they want to take on themselves.

However, the harm-based reason for a legal—and, less controversially, a moral—obligation to be vaccinated is grounded in considerations of risk of harm to others, which apply both to children and adults. Arguably, it is an individual’s, a parents’, and a state’s responsibility to take reasonable steps to prevent individuals from posing serious risks of harm to others.

Jessica Flanigan^36^ has argued that failure to vaccinate should be prohibited—that is, that vaccination should be compulsory—on the basis of harm prevention, in the same way as people are prevented from randomly firing a gun in an open space. An infectious disease is in this respect like a bullet that can be shot and harm or even kill innocent people. Another analogy that has been used to support the same point is that of allowing children to go around with a bottle of a toxic substance like bleach that, if accidentally spilled, could cause serious harm or even death.^37^ Once again, in the same way as we legitimately prohibit children from doing that, we should prohibit children from going around risking ‘spilling’ viruses. Considerations of harm prevention prevail over considerations of individual liberty. The second analogy seems more apt because it better reflects the situation with regard to vaccination and infectious disease. Unlike the gunfiring example, in the bleach example the harm or risk of harm posed on others is unintentional, but this does not mean that there no good reasons to prevent it. Preventing harm or risk of harm to others is normally taken to be a sufficient ground for liberty restrictions, at least when the cost on individuals of preventing risk of harm to others is small.

Of course, considerations of harm prevention through vaccination need to be weighed against the consideration of the small risks of vaccines. Some have argued that vaccination should not be mandatory when there is herd immunity, because vaccinating individuals in that case would not reduce risks for the population, but would expose individuals to the however small, but unnecessary risks of vaccines.^13^ Thus, when there is herd immunity, harm prevention considerations would weigh against vaccination. However, this kind of risk assessment would also need to factor in the risk that immunization rates drop below the threshold of herd immunity (as they often do), which would reintroduce the risks of infectious diseases for the unvaccinated. Besides, other ethical considerations beyond risk prevention are sometimes taken to weigh in favour of exposing individuals to the very small risks of vaccines even in presence of herd immunity. These are considerations of fairness.

## Fairness

A second ethical issue has to do with the nature of ‘public good’ of herd immunity. Public goods raise ethical issues on their own that are quite independent from the considerations of individually imposed risk of harm. In particular, public goods normally raise issues of fairness related to the freeriding problem. In many cases, everyone is expected to contribute to important public goods even if doing or failing to do so does not make a difference in terms of significantly decreasing or increasing risk of harm to others, and even if for any individual it would be better not to contribute. If I use public transport, I am expected to pay the ticket even if it does not make difference to the efficiency of the service and even if I would be better off without paying, as long as enough others do so. The same can be said about herd immunity. I would be better off by not being vaccinated, as long as enough others do so and guarantee herd immunity; in such case my non-vaccination would not make a significant difference to the level of risk I impose on others, and I would still be indirectly protected without having to take on myself the small risk or the annoyance of getting vaccinated. Thus, there are two kinds of fairness requirements: (1) a requirement not to free-ride on a public good like herd immunity, when the public good exists; and (2) a requirement to make one’s contribution to an important public good like herd immunity, regardless of whether the individual contribution ‘makes a difference’.^15^

Fairness is sometimes taken to be a reason for a moral obligation to get vaccinated against certain infectious diseases,^34^ in addition to considerations about harm prevention. On some other views, drawing on analogies with other ways in which fair contribution to important public goods is made compulsory (e.g. taxation^15^), fairness is a strong enough reason to make vaccination compulsory quite independently of whether it ‘makes a difference’ and on whether a community already has herd immunity.

## Conclusion

The ethics of vaccination exemplifies very well the interdependence of individual responsibilities (e.g. to be vaccinated), collective responsibilities (e.g. to realize herd immunity against infectious diseases) and institutional responsibilities (e.g. to enact policies that guarantee herd immunity, for instance mandatory vaccination, or that guarantee that herd immunity is realized fairly). Here, I have provided a general overview of the main ethical issues involved, but of course more specific ethical issues are raised by certain vaccines and do not apply to all vaccines. For example, evidence suggests that the flu vaccine is more effective at building up immunity at the collective level if targeted at children, even if those who benefit the most from protection from the flu are the elderly. In this case, some have argued that whatever vaccination policy we adopt, it should target children primarily in order to maximize the benefit of the vaccine.^38^

Another factor that affects the strength of the ethical considerations above is the effectiveness of a vaccine at stopping transmission. Again, this is relevant with regard to the future COVID-19 vaccine. There is a realistic possibility that what currently is the most promising vaccine candidate, being developed at Oxford, will be effective at preventing people from getting sick but not too effective at blocking transmission (this hypothesis is at the moment based on observations on animal models). If so, the cases for compulsory vaccination based on fairness and of harm prevention seem to be weaker—although they might still be supported on the basis of the high toll that COVID-19 infections are imposing on healthcare systems.

While research ethics, and the ethics of vaccination research specifically, is a broader area that falls outside the scope of this article, it is worth mentioning that the ethics of vaccination decisions and vaccination policies is not independent of it. The way vaccines are researched and developed can affect policies. For example, vaccines obtained using cell lines derived from aborted foetuses raise the issue of whether people with certain religious or moral views around abortion should be exempted from vaccination mandates. Or, if vaccine research needs to be fast-tracked to address emergency, as is the case with the COVID-19 vaccine candidates currently being trialled, there might be some more uncertainty around its possible side effects when rolled out at the population level, which in turn can weaken the ethical case for vaccine mandates.

Infectious diseases have always been present in human history. The COVID-19 pandemic is a good indicator of the fact that they are always going to be present. It is therefore vital that the interplay between individual, collective and institutional responsibilities with regard to vaccination decisions and policies remains at the centre of future philosophical, sociological and legal work on vaccination.

## Data Availability

*No new data were generated or analysed in support of this research.*
